# Vicarious Menstruation

**Published:** 1852-12

**Authors:** 


*Vicarious Menstruation.* By A. W. Knight, M. D., of White Springs, Hamilton Co., Florida.

The following case may, perchance, interest some of your readers, or, at least, it will go to prove our text-books correct, when they assert that an evacuation may take place at various parts of the body, vicarious of the normal uterine periodical secretion.

On the 8th of February of the present year, I was called to visit Mrs. W., of Columbia county, who was much alarmed at a hæmorrhage from the *left eye:* she is about thirty years of age, and the mother of six children. Reported to me, that for four or five nights past, she had perceived that her left eye had been bleeding about a tablespoonful during the night, as near as she could judge, from the stain upon the pillow, and that the eye was suffused with blood during the day. During the same period, she had a slight hæmorrhage from the nose, and had discovered traces of blood in the expectoration. Feeling no pain in the eye, and having never received any injury from blow or otherwise, she was at a loss how to account for it.

Making particular inquiries into the case, I learned that she had menstruated in the usual manner only three times in twelve years, and that this was the proper time for the menses to appear. Her general health, during this time, has not been good, and her appearance, at the time of my visit, was decidedly anæmic, with some tendency to anasarca.

On careful examination of the eye-lid, I could detect some slight congestion of the minute blood-vessels; otherwise not different from the other. With a view to the improvement of her general health and to the re-establishment of the uterine function, I prescribed mineral tonics, chiefly the preparations of iron, for the interval, and special emmenagogues for the week on which the menses ought to return. Up to the present month she has had no return of the vicarious menstruation.

She followed my prescription for about one month; but the normal discharge not taking place, she became discouraged, and discontinued the medicine. Having understood that, some years since, her menses appeared after a few days' bathing in the sulphur spring at this place, and as there has been no return of the regular menstruation, I have advised her to try the bathing again.

A few weeks since, I was called to treat her for intermittent fever, and was struck with a remark of her's—that the side on which the vicarious discharge appeared "felt *numb* when the fever was on." Does this throw any light upon the state of the nervous system in "intermittents?" Could the peculiar condition of the uterine system cause any loss of sensation in the nerves of one side more than the other? Here is a point worthy of investigation by older practitioners than myself.

The "intermittent" yielded readily to treatment; and once since the convalescence she says that symptoms have induced her to think, that the vicarious discharge may again return from the eye.

August 24, 1852.—*Southern Med. and Surg. Journal.*

